# Comprehensive assessment of myocardial mechanics in mice using 3D cine DENSE

**DOI:** 10.1186/1532-429X-13-S1-P30

**Published:** 2011-02-02

**Authors:** Lauren B Gibberman, Xiaodong Zhong, Andrew D Gilliam, Craig H Meyer, Brent A French, Frederick H Epstein

**Affiliations:** 1 University of Virginia, Charlottesville, VA, USA; 2 Siemens Healthcare, Atlanta, GA, USA

## Introduction

MRI of myocardial mechanics in mice enables the investigation of the roles of individual genes and experimental therapies in cardiac function. While two-dimensional tagging, HARP, and DENSE have previously been demonstrated for the mouse heart, myocardial mechanics are more comprehensively assessed using three-dimensional (3D) methods.

## Purpose

We sought to develop 3D cine DENSE acquisition and analysis methods and to evaluate them for measuring 3D mechanics in normal mice.

## Methods

A multiphasic volumetric spiral cine DENSE sequence with 3D displacement encoding was implemented on a small-bore 7T MRI system (Clinscan, Bruker). Magnitude and phase images were reconstructed online, and semiautomatic segmentation methods followed by automatic strain, twist, and torsion calculations were implemented offline. Seven healthy C57Bl/6 mice were studied. For imaging, mice were anesthetized with 1.25% isoflurane, positioned in a 32 mm diameter birdcage RF coil, and maintained at 36°C. The ECG was monitored and used to trigger data acquisition. After localizer imaging, 3D cine-DENSE imaging was applied. DENSE sequence parameters included voxel size = 0.25 x 0.25 x 0.4 mm^3^, number of partitions = 21, TR = 7 ms, and number of cardiac phases = 14. The entire left ventricle (LV) was covered in a scan time of approximately 23 minutes.

## Results

**Figure 1:**
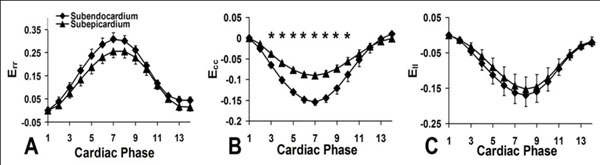
Normal midventricular subendocardial and subepicardial strains (A: E_rr_, B: E_cc_, C: E_ll_) measured by 3D cine DENSE in 7 mice:.

**Figure 2:**
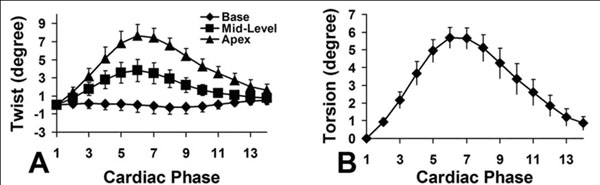
Normal LV twist and torsion measured by 3D cine DENSE.

Volumetric cine DENSE data covering the entire LV of all 7 mice were analyzed. Semiautomatic segmentation took approximately 1 hour per mouse. Three-dimensional mechanics were quantified throughout the LV, including the 3D strain tensor (E_rr_, E_cc_, E_ll_, E_rc_, E_rl_, and E_cl_), twist, and torsion. Average mid-ventricular strain-time curves for the 3 normal strains are shown in Figure 1. All strain values are in close agreement with previous studies. Myocardial twist and torsion are shown in Figure 2. Although not shown, all three shear strains were measured and are similar to previous measurements in humans.

## Conclusions

Using these data acquisition and analysis methods, a comprehensive assessment of 3D myocardial mechanics in mice can be performed with a scan time of less than 25 minutes and a segmentation time of about an hour. In future studies, off-resonance blurring of spiral images could be reduced using better shimming or deblurring methods. These techniques may find application for evaluating the effects of experimental therapies and for phenotyping genetically-engineered mice.

